# High Velocity Suspension Flame Spraying (HVSFS) of Metal Suspensions

**DOI:** 10.3390/ma13030621

**Published:** 2020-01-30

**Authors:** Matthias Blum, Peter Krieg, Andreas Killinger, Rainer Gadow, Jan Luth, Fabian Trenkle

**Affiliations:** 1 Institute for Manufacturing Technologies of Ceramic Components and Composites (IMTCCC), University of Stuttgart, Allmandring 7b, 70569 Stuttgart; Germany; 2 obz innovation GmbH, Elsässer Straße 10, 79189 Bad Krozingen, Germany

**Keywords:** high-velocity suspension flame spraying, copper, silver, NiCr 80/20, metal coatings

## Abstract

Thermal spraying of metal materials is one of the key applications of this technology in industry for over a hundred years. The variety of metal-based feedstocks (powders and wires) used for thermal spray is incredibly large and utilization covers abrasion and corrosion protection, as well as tribological and electrical applications. Spraying metals using suspension- or precursor-based thermal spray methods is a relatively new and unusual approach. This publication deals with three metal types, a NiCr 80/20, copper (Cu), and silver (Ag), sprayed as fine-grained powders dispersed in aqueous solvent. Suspensions were sprayed by means of high-velocity suspension spraying (HVSFS) employing a modified TopGun system. The aim was to prepare thin and dense metal coatings (10–70 µm) and to evaluate the process limits regarding the oxygen content of the coatings. In case of Cu and Ag, possible applications demand high purity with low oxidation of the coating to achieve for instance a high electrical conductivity or catalytic activity. For NiCr however, it was found that coatings with a fine dispersion of oxides can be usable for applications where a tunable resistivity is in demand. The paper describes the suspension preparation and presents results of spray experiments performed on metal substrates. Results are evaluated with respect to the phase composition and the achieved coating morphology. It turns out that the oxidation content and spray efficiency is strongly controlled by the oxygen fuel ratio and spray distance.

## 1. Introduction

In recent years the high-velocity suspension flame spraying (HVSFS) process, an evolution of the well-established HVOF process that allows for spraying of liquid feedstock instead of finely grained spray powders, has been developed. Whereas the majority of studies focus on the spraying of oxide ceramics, such as aluminum oxide [1,2], titanium oxide [3,4], or zirconium oxide [5], glasses [6], and biomaterials [7,8], there are not many studies on the topic of suspension sprayed metal coatings. Among these, more focus can be found on the topic of suspension plasma spraying [9,10], while only few publications cover the suspension flame spraying of metals, for instance Inconel alloys [11].

The use of suspensions in the flame spraying process allows for the processing of submicron- and nanopowders to form finely structured coatings with reduced coating thickness, which can lead to an improvement of the mechanical, thermal and chemical properties of the coatings [12]. While suspension flame spraying offers many advantages, it also increases the complexity of the process due to the additional parameters (such as suspension formulation) that have to be considered. In the case of metals, the suspension formulation is especially important and challenging due to the surface reactivity and high density of the involved metal powders.

In this study, suspensions from three metals were prepared and sprayed via HVSFS: NiCr, silver, and copper. While powder and wire spraying of NiCr alloys and Cu is more common in thermal spraying, Ag has not been in the focus; and to our knowledge, none of these materials have been applied in suspension spray processes so far. The aim of this study is to create metal coatings with a reduced thickness in the range of 10–50 µm. The amount of oxides in the coating should be as low as possible, especially for Cu and Ag. For Ag, a high deposition efficiency is also mandatory to keep material costs in a reasonable range.

NiCr coatings are mostly used as protective coatings or for heating elements and can be sprayed by various thermal spray techniques [13,14]. While copper can also be deposited by different thermal spray techniques, cold gas spraying of copper offers low oxidation and porosity, which results in good electrical properties of the coatings [15]. Silver is a less common material in thermal spraying. There are only very few studies on the cold gas spraying of silver or silver composites for electrical contacts [16,17]. Silver is also well known for its antibacterial effect. Thin suspension-sprayed coating may also serve as an antibacterial layer in medical applications.

Ultrafine Ag powder can also be incorporated as a dopant in biomedical coatings to display their antibacterial properties. This has been examined in recent studies for different calcium phosphate- based coatings. One route uses HVSFS with mixed Ag/HAp, TCP, or bioglass containing suspensions [18], the second route uses a standard plasma spraying process with agglomerated powders containing Ag particles [19].

As already mentioned, one of the main competing spray method for spraying soft metals like copper or silver is cold gas spraying (CGS). A comparative study with CGS has been recently published by some of the authors [20]. Copper and silver powders were sprayed using a Kinetiks 4000 system (CGT Cold Gas Technology GmbH, Ampfing, Germany). As powder feeder operated spray systems are restricted to a minimum powder grain size of >5 µm (typically grain size distribution in the range of 5–25 µm) coatings show higher roughness and thickness than suspension sprayed coatings, but it is well known that CGS produces a more or less pore-free coating structure when well-plasticizing metals, like aluminum, copper, or silver, are used as a feed stock. Due to the coarser spray particles, CGS coatings are usually limited to a minimum thickness of approx. 20 µm, but are theoretically not limited in regard to a maximum thickness value. In contrast, HVSFS can go below 10 µm, but is limited at higher thickness due to the increasing formation of residual stresses in the coating when increasing the coating thicknesses. As CGS is operated on a significantly lower thermal level of approx. 800 °C, the amount of oxides in the coating can be usually kept significantly lower than in HVSFS coatings. This is especially relevant for electrical applications, where a high electrical conductivity is demanded. Therefore, spraying copper is one of the main industrial application in CGS industry. In any case, a more elaborate comparative study is necessary to compare electrophysical properties of Cu and Ag coatings sprayed with the two methods.

## 2. Materials and Methods 

### 2.1. Preparation and Characterization of the Metal Suspensions

**Figure 1:**
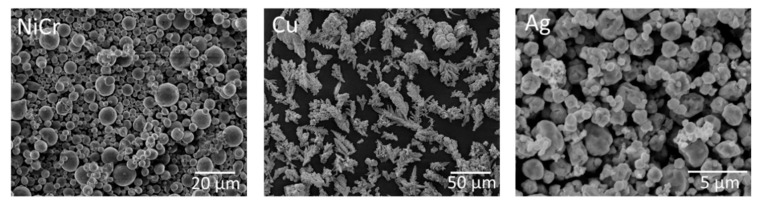
SEM images of the metal powders used in this study.

Three different metal powders were selected for this study. Available manufacturer data have been summarized in Table 1. The first was a gas atomized NiCr powder (H.C. Starck, Goslar, Germany), the second was a chemically derived copper powder (Evochem GmbH, Offenbach, Germany) and the third was an agglomerated silver powder (Metalor Technologies SA, Neuchatel, Switzerland). As can be seen in Figure 1, the NiCr powder shows a perfect spherical structure, which is generally achieved in the gas atomization process. The agglomerated and sintered silver powder also shows a more or less spherical structure, whereas the copper particles show a dendritic and irregular structure, resulting from the chemical precipitation process.

The materials were dispersed in deionized water with a solid content of 18 wt% for the copper suspension and 30 wt% for the NiCr and the silver suspension. In contrast to organic solvents like isopropanol or ethanol, deionized water does not deliver combustion enthalpy and the evaporation enthalpy is significantly higher (water: 2.26 kJ/g; isopropanol: 0.66 kJ/g; ethanol: 0.84 kJ/g). Therefor it was used as dispersant to reduce the total combustion enthalpy, leading to a reduction in flame temperature and thus reducing oxidation of the metal particles. To improve the stability and to prevent sedimentation and clogging during the process, suspensions were stabilized using an organic dispersant, an anti-foaming agent and a rheology additive. The amount of solid content has been chosen to adjust the viscosity behavior of the suspensions. In case of copper, the solid content could not be increased to higher value due to the irregular particle morphology that promotes clogging in the feedline. It has been described in more detail in a previous publication [21]. The particle size distribution was measured by laser diffraction (Mastersizer 3000, Malvern Instruments, Malvern, UK). The rheological properties of the suspensions were characterized using a MCR 302 rheometer (Anton Paar GmbH, Graz, Austria). The measurements were performed at 25 °C using a double-gap cylinder.

### 2.2. Coating Deposition and Characterization

For the HVSFS process, a modified high-velocity flame spray torch type TopGun (GTV GmbH, Luckenbach, Germany) was employed. In the modified torch, the powder injector is replaced with a suspension injector unit, which allows for injection of the suspension axially into the combustion chamber. The suspension injector is equipped with a simple turbulence nozzle having an orifice diameter of 0.5 mm. A more detailed description of the HVSFS process can be found in [12].

The coatings were deposited on planar stainless steel (X6CrNiMoTi17-12-2) substrates. In case of some Ag-coatings pure copper samples were alternatively used as substrate. Dimensions of all substrate samples were 50 × 50 × 3 mm. Surface activation was done by grit-blasting using F120 corundum at a pressure of 5 bar. Prior to coating operation, all specimens were cleaned with acetone. Whereas the TopGun-system allows for the use of a wide variety of fuel gases, all coatings in this study were sprayed using an ethene-oxygen mixture. The torch was operated either at a stoichiometric ethene-oxygen ratio (denoted as λ = 1) and, for comparison also at different sub-stoichiometric ratios, thus having a surplus of ethene (denoted as λ = 0.57; 0.63, and 0.87, respectively). The idea in mind is to have a reducing flame chemistry that may reduce oxidation of metal particles in the spray process.

The torch was operated on a six-axis robot describing a meander movement in front of the planar substrates with a torch speed of 500 mm/s. Two air cooling nozzles were mounted on the torch to simultaneously cool the substrate during spray process using compressed air. The two cooling spots are located on both sides near the spray focus on the substrate surface. Samples were sprayed at five distances: 60 mm, 75 mm, 90 mm, 105 mm, and 120 mm and a suspension feed rate of 40–55 g/min. An overview of the spraying parameters can be found in Table 2. It should be noted that for all samples a fixed amount of torch passes was used (NiCr and Cu: eight passes; Ag: four passes) also holding constant the suspension feed rate for each system. This allows for comparison of relative deposition efficiencies assuming comparable porosity values for all coatings. In case of HVSFS coating porosity typically is in the range of 0–5%. Thus, when calculating DE from coating thickness the error is in the same magnitude (0–5%). In the case of silver, the deposition efficiency was measured following DIN EN ISO 17836 according to Equation (1):

(1) $$DE\;\left[\%\right]=\frac{m_{c}}{m_{t}}\times 100$$

m_c_: mass of coating
m_t_: theoretically sprayed mass

Further spray parameters that are listed in Table 2 are: ethene-oxygen ratio λ, total gas flow rates (TGFR), separate gas flow rates (GFR) for ethene and oxygen. Spray distance variations, meander offset and torch speed have identical values for all three materials.

Cross-section images of the coatings were taken using a Leica MEF4M (Leica GmbH, Wetzlar, Germany). The coating thickness was measured on eight different points of these cross-sections.

The surface structure of the coatings was investigated using a white light interferometry (Bruker ContourGT-K, Bruker, Mannheim, Germany) using a 5× objective. Three surface areas with a size of 1.2 × 0.9 mm were evaluated.

A comparative microhardness measurement study was performed to analyze the coating properties for the different spray parameters, i.e., spray distance, λ, and total gas volume. Vickers microhardness (HV_0.1_) measurements were carried out on polished cross-sections using a Fischerscope H100 instrument (Helmut Fischer, Sindelfingen, Germany). A set of 20 measurements were performed on one probe and evaluated statistically. The study was made in accordance to DIN EN ISO 12577.

High-resolution SEM pictures and the EDX measurements were performed using a SEM DSM 982 Gemini (Zeiss AG, Oberkochen, Germany).

XRD analysis on coatings have been carried out using a X´Pert MPD diffractometer using Cu Kα (40 kV/40 mA) in combination with a Panalytical X´Celerator detector (both from Panalytical GmbH, Herrenberg, Germany). The used database was taken from International Centre for Diffraction Data (JCPDS ICDD). For qualitative analysis “Crystallographica Search Match” software (Oxford Cryosystems Ltd., Long Hanborough, UK) has been used. The Siroquant software (Sietronics Pty Ltd., Mitchell, ACT 2911, Australia) was used for quantitative Rietveld analysis.

## 3. Results and Discussion

### 3.1. Suspension Characterization

**Figure 2:**
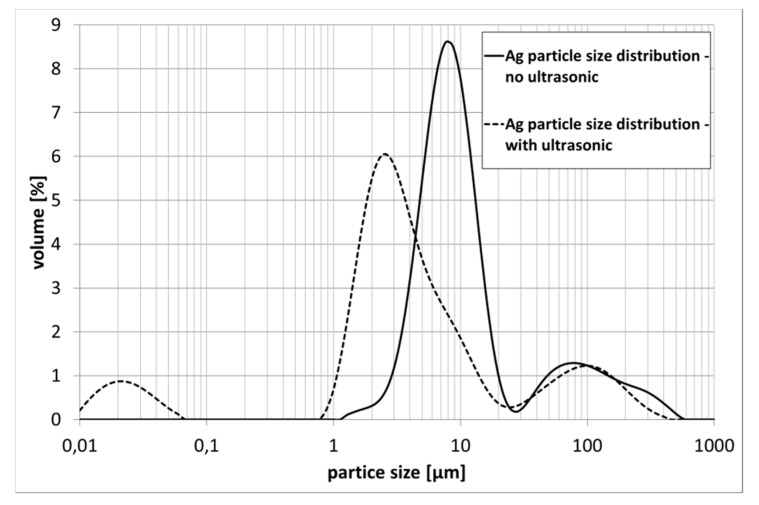
Particle size distribution of silver suspension—with and without ultrasonic treatment.

Suspensions were evaluated regarding their particle size distribution, their sedimentation behavior and their rheological properties. It was found, that particles tend to form agglomerates in the suspension, which can be redispersed using ultrasonic treatment. In Figure 2, the particle size distribution of the stabilized silver suspension is displayed. If the sample is treated ultrasonically, the measured particle size is reduced significantly. However, treatment of the suspension using ultrasonic has to be done carefully, as it degrades the stabilization of the suspension, which leads to a faster sedimentation of the suspended particles.

By applying ultrasonic treatment, average particle size d50 in silver suspension drops from 9.5 µm to 3.6 µm. This effect can also be observed for the copper suspension, showing a measured average particle size d50 of 64.8 µm without, and 34.6 µm with, ultrasonic treatment. Especially, agglomerated powders show a weak mechanical cohesion and easily form fragments. This effect is even stronger when applying ultrasonic treatment. The NiCr suspension does not show this effect (d50 = 10.8 µm). This can be mainly explained by the powder particle properties. The gas atomized particles are mechanical stable and do not tend to break.

**Figure 3:**
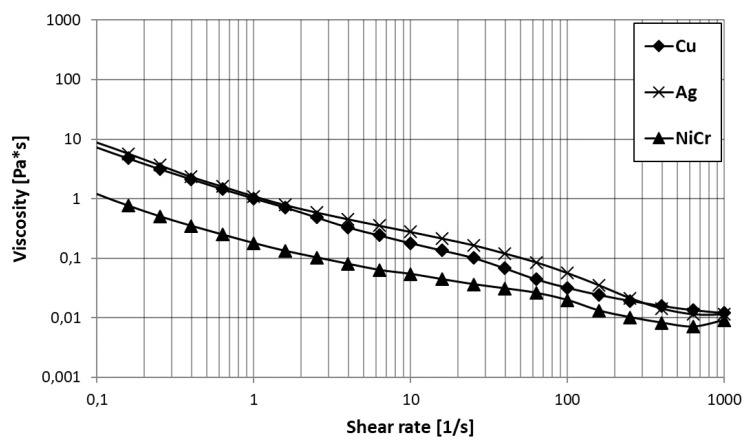
Flow curves of the Cu, Ag, and NiCr suspensions used in this study.

The flow curves of the suspensions are shown in Figure 3. All three suspensions show shear thinning behavior. The observed difference between the NiCr suspension and the Ag and Cu suspensions is probably due to a slightly lower concentration of rheology additive in the NiCr suspension. Particle morphology also has an effect on rheological behavior of the suspension.

### 3.2. Coating Structure and Properties

#### 3.2.1. NiCr Coatings

**Figure 4:**
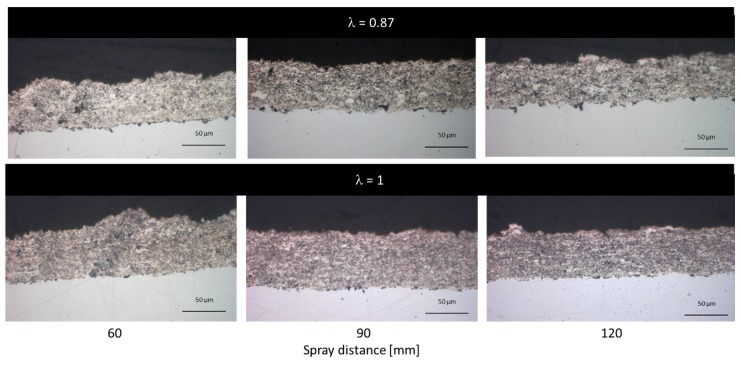
Optical microscope cross-section images of NiCr coatings sprayed with two different λ (0.87 and 1) and with three different spray distances (60, 90, and 120 mm).

In Figure 4, a collection of cross-sections of HVSFS sprayed NiCr coatings at different spraying distances and oxygen-to-ethene ratios are shown, the bright areas correspond to metal-rich, dark areas to oxide phases.

**Figure 5:**
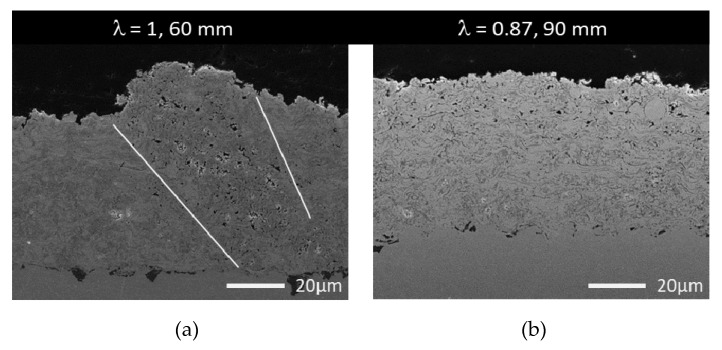
SEM image of NiCr coatings. (**a**) A typical cone-shaped defect found in NiCr coatings at 60 mm spray distances (λ = 1). (**b**) Spray parametres: λ = 0.87 (<1); spray distance = 90 mm. Taken from [21].

**Figure 6:**
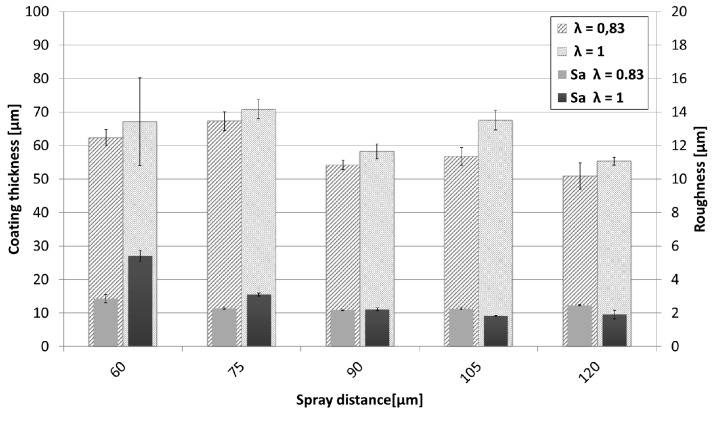
Comparison of coating thickness and roughness values of the NiCr coatings sprayed at different spray distances and λ values.

At a very close spraying distance of 60 mm, an increased formation of defects can be observed. The formation of these defects begins near the substrate and increases in size during the build-up of the coating, resulting in a cone-shaped defect. Fauchais et al. described the formation and possible reasons of this defects [22,23,24]. These defects grow at an angle from the surface and show an increase in porosity (Figure 5). Consequently, the surface roughness Sa (shown in Figure 6) of these coatings is significantly higher. This effect is more pronounced at a stoichiometric oxygen-to-ethene ratio.

The variation of the oxygen-to-ethene ratio λ clearly affects the oxidation of the NiCr particles. The coatings sprayed at a surplus of ethene (λ < 1) contain more metallic particles (visible as the bright phase in the light micrograph), while the coatings sprayed at stoichiometric parameters show higher oxidation (darker phases visible in the micrographs in Figure 4). A similar approach is described in detail by Förg et al. in the case of suspension spraying of Cr_3_C_2_. Varying λ allows for an adjustment of the formation of Cr_2_O_3_ [25]. Consequently, for NiCr coatings, it is possible to adjust the electrical conductivity and resistance as demanded by the desired application (i.e., heating elements) [26].

As all coatings have been prepared using the same amount of spray passes (= 8), the achieved coating thickness can serve as an indicator for deposition efficiency. These are summarized in Figure 6 together with the Sa values. Deposition efficiency slightly rises with decreasing spray distance. When spraying under stoichiometric conditions (λ = 1), slightly higher deposition efficiencies can be observed. As already mentioned, at lower spray distances more coating defects occur that contribute to a significantly increased surface roughness.

**Figure 7:**
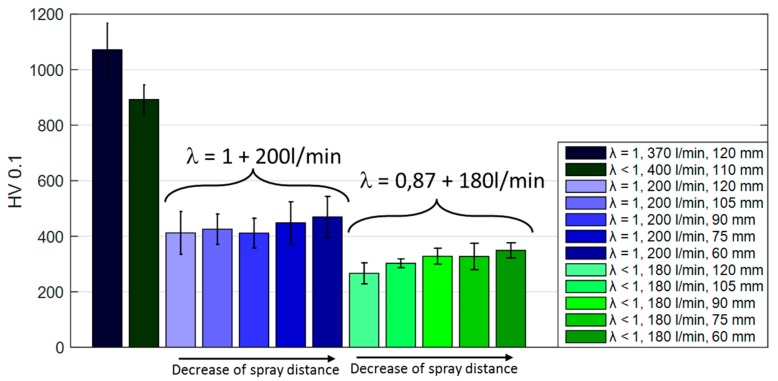
Comparison of Vickers hardness HV0.1 for NiCr coatings sprayed with different λ (0.87 and 1) and at different spray distances (60; 75; 105; 110; 120 mm).

The microhardness measurement results are summarized in Figure 7. Hardness values differ significantly and show a dependency of spray distance, λ and total gas volume. For lower gas volumes (180 and 200 L/min), the spray distance clearly controls hardness values. As observed in the microscope images higher spray distances show higher porosities and correspondingly lower hardness values. On the other hand, a higher λ together with a slightly higher total gas volume increases oxidation significantly. Doubling the total gas volume doubles the hardness value. Both factors may contribute: the higher kinetic impact leads to a denser coating structure thus increasing the hardness; the significantly increased heat impact on the other hand leads to a strong increase in oxide species which in turn will increase hardness.

**Figure 8:**
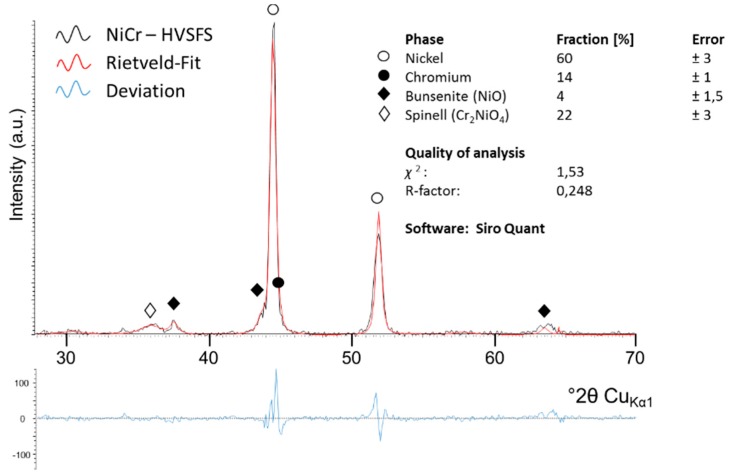
Representative XRD spectrum of a HVSFS sprayed NiCr coating. A Rietveld fit has been applied to the spectrum. Spray parameters (λ = 0.87; total gas flow: 180 L/min; spray distance: 90 mm).

XRD analysis has been carried out to analyze type and quantity of the oxide phases. The NiCr coating sprayed with λ = 0.87 (also noted as λ < 1 in Figure 7) and a spray distance of 90 mm was analyzed for this purpose, please refer to Figure 8. Based on the Rietveld method, a quantitative analysis was performed to learn more about the oxidation species that form during spraying. Achieved results are summarized in Table 3. Additionally, to the metal phases, two major types of oxides can be found in the coating: Spinel (Cr_2_NiO_4_) and bunsenite (NiO). From this data it can be concluded that the oxide fraction in the coating is in the range of 25%–30%.

**Figure 9:**
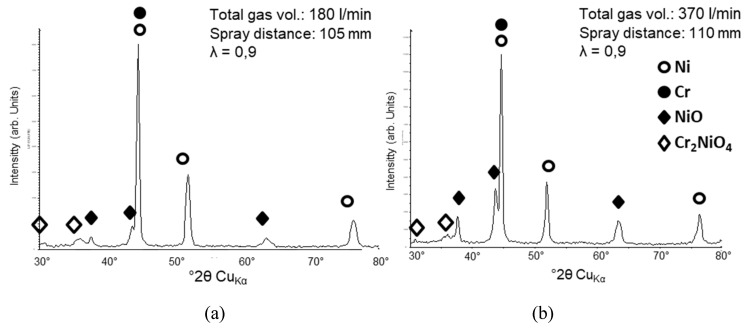
Comparison of XRD spectra for NiCr coatings sprayed with two different total gas flow amounts (180 vs. 370 L/min) as noted in the graph. λ was kept constant, and the spray distance differs slightly. ♦ symbols highlight the most prominent oxide peaks that were identified according to Figure 8.

A comparison of the oxide content for two different total gas flows (180 vs. 370 L/min) shows a significant increase of the oxide peaks in XRD. An increase of the total gas flow from 180 to 370 L/min leads to a doubling of the heat flux as well as a strong increase of the turbulence on the surface. This leads to a higher substrate temperature and thus to higher oxidation rates. Additionally, the slightly higher spraying distance could have an influence. In Figure 9, both spectra are shown for comparison. The relevant oxide peaks are significantly increased. A quantitative Rietveld evaluation estimates the amount of oxide above 50%.

#### 3.2.2. Cu Coatings

**Figure 10:**
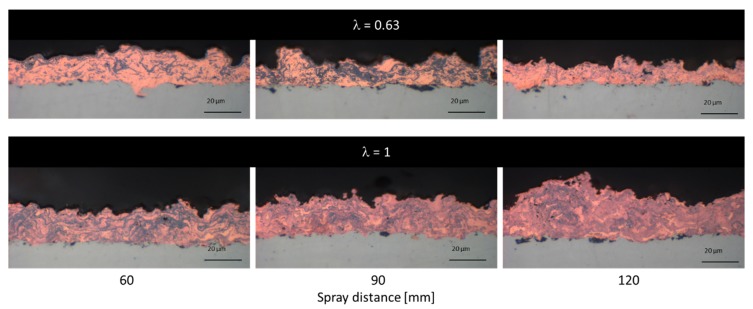
Optical microscope cross-section images of the Cu coatings sprayed with two different λ (0.63 and 1) and with three different spray distances (60, 90, and 120 mm). Taken from [21].

The cross-sections of the copper coatings, as shown in Figure 10, show the difference in oxidation between the stoichiometric and the sub-stoichiometric parameters. At λ < 1, there are distinguishable metallic and oxide layers, which are more pronounced at closer spraying distances. This is presumable due to the higher heat flux at close distances, resulting in a post-oxidation process of the surface layer of the coating.

In contrast, the coatings sprayed at stoichiometric ratios show a more homogeneous distribution of oxide phases. While the increased heat flux at close distance will also influence the oxide formation, the dominating mechanism here appears to be the in-flight oxidation of the particles during the spraying process. The cross-sections show dense microstructures for all copper coatings with low porosity, suggesting that a sufficient melting of the particles during the spraying process has occurred.

**Figure 11:**
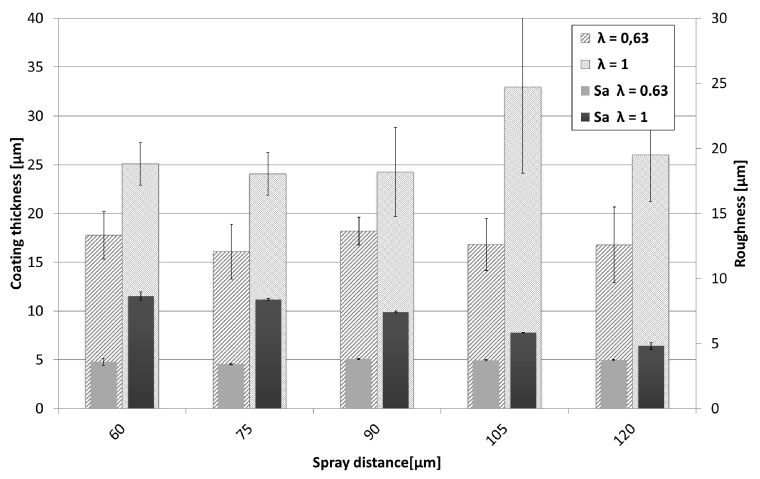
Comparison of coating thickness and roughness values of the Cu coatings sprayed at different spray distances and λ values.

As already discussed in the previous section, the achieved coating thickness again can serve as an indicator for deposition efficiency. Coating thicknesses and surface roughness of the Cu coatings, as shown in Figure 11, indicate slightly higher deposition efficiencies for the stoichiometric parameters as well as higher values for the surface roughness, which decreases with increasing spraying distance. Interestingly, when directly compared to NiCr, the spray distance seems to have a minor influence on these values.

**Figure 12:**
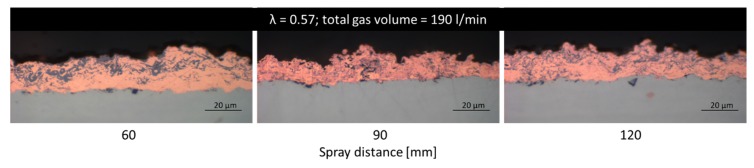
Optical microscope cross-section images of Cu coatings sprayed with a reduced of λ = 0.57 with increasing spray distance (from left to right). Applied spray parameters: λ = 0.57; total gas volume = 190 L/min.

For comparison a set of samples were sprayed with a reduced λ of 0.57, because it was observed that oxidation can be further reduced. Interestingly, oxidation now occurs more in the outer part of the coating, and is somewhat lower near the interface. Respective coatings are displayed in Figure 12.

**Figure 13:**
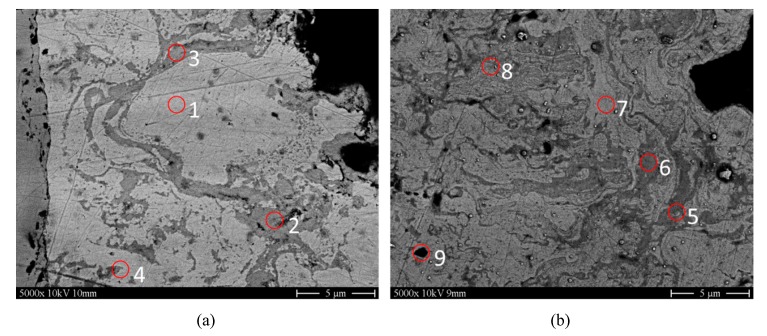
SEM images of two Cu-coatings prepared for EDX-analysis. Left: (**a**) λ = 0.57; total gas volume = 190 slpm; Right: (**b**) λ = 1; total gas volume = 230 slpm. Taken from [21].

As oxidation is clearly visible in all samples, EDX analysis was performed to analyze the distinguishable phases in regard of their Cu and O content for to Cu coatings at different λ (0.57 and 1) and two different total gas volumes (190 and 230 slpm); refer to Figure 13. For both Cu coatings, a higher amount of oxygen can be detected in the dark phase (6–7 wt%). The bright phase is more pronounced in sample A (λ = 0.57; total gas volume = 190 slpm) with low oxygen content (0.5–1 wt%), whereas in sample B (λ = 1; total gas volume = 230 slpm) metal and oxide lamellas are more densely mixed and cannot be clearly separated by EDX. Thus, the oxygen content of the brighter phase is measured at a higher level (between 2–3 wt%). However, the polished surface contains a high amount of impurities, which is due to the somewhat difficult conditions when preparing cross-sections of the smooth copper material in metallography (noted as other in Table 4). There is a high risk of working in impurities into the polished surface during the polishing process.

**Figure 14:**
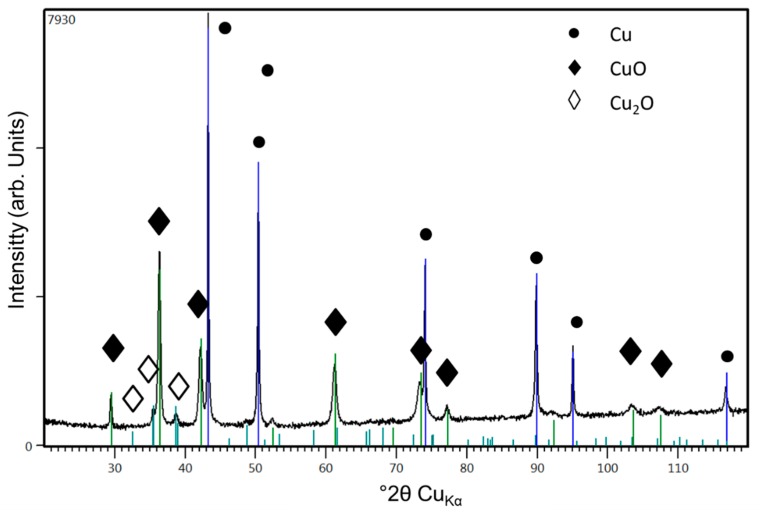
XRD spectrum of HVSFS sprayed Cu coating shown in Figure 12 on the left (λ = 0.57; spray distance = 60 mm and total gas volume = 190 l/min). CuO and Cu_2_O. Strongest peaks are labeled.

XRD analysis of HVSFS sprayed copper shows clearly distinguishable peaks for metallic Cu, and oxide species namely copper(II)oxide (cupric oxide, CuO) and copper (I) oxide (cuprous oxide, Cu_2_O). Figure 14 shows the spectrum for a coating with spray parameters identical to those shown in Figure 12, left (λ = 0.57; spray distance = 60 mm and total gas volume = 190 l/min).

#### 3.2.3. Ag Coatings

**Figure 15:**
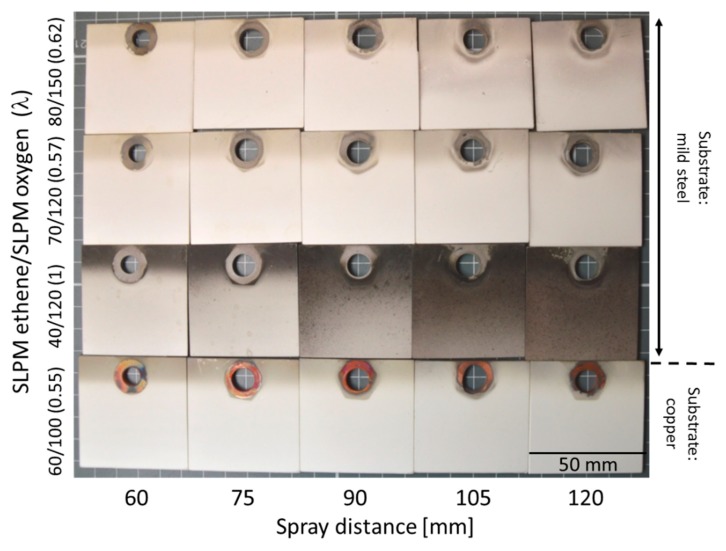
Color scheme of silver coating surfaces sprayed at different parameter sets worked out in a pre-study. Size of each sample is 50 × 50 mm.

When spraying silver coatings, it was observed, that in order to achieve a suitable coating, the appropriate parameter window appears to be much narrower than for NiCr and copper. In a parameter pre-study, coatings have been sprayed on mild steel and on copper substrates as can be seen in Figure 15. The colors of the coatings after spraying differ significantly depending on what spray distance and what λ has been chosen. Colors change from brown, yellow to a bright white. This behavior is probably due to formation of different amount of oxide species in the coatings.

**Figure 16:**
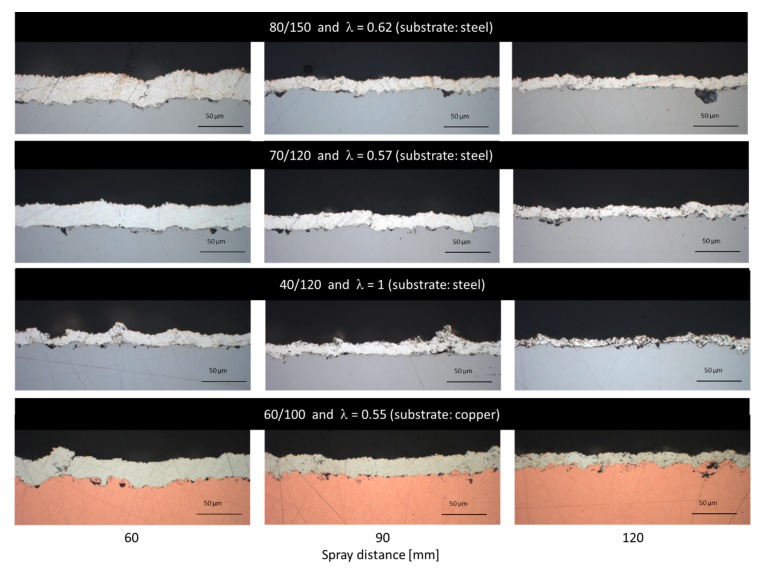
Optical microscope cross-section images of the Ag coatings on mild steel and Cu substrate from samples in Figure 15 chosen for 60, 90, and 120 mm spray distances. Please note that in respect of gas ratios and substrate type, images are organized in the same systematic as in Figure 15.

Respective cross-sections of the coatings for spray distances at 60, 90, and 120 mm are shown in Figure 16. The stoichiometric parameters yield a lower coating thickness and, consequently, a lower deposition efficiency at all spraying distances. Due to the higher spraying distance, the residual time of the particles in the flame is increased and the formation of oxide species may be favored. As a consequence, the coating shows a more and more greyish color at higher spray distances compared to the bright color at closer ones.

**Figure 17:**
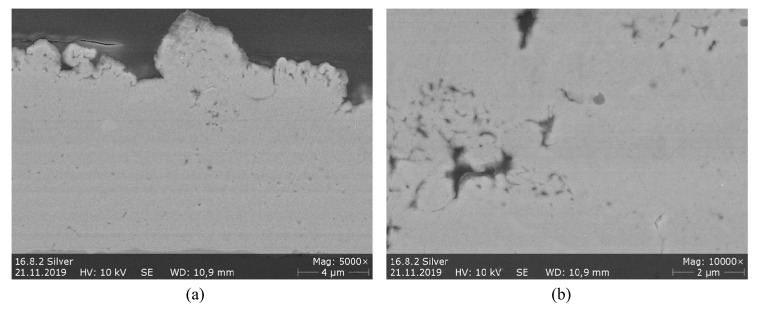
SEM images of Ag-coating on copper substrate with parameter set 60/100, λ = 0, 55, 90 mm, (refer to Figure 16 last row). (**a**) near surface (**b**) details of pore structure.

The cross-sections, as well as the color of the coatings, suggest that best results are achieved at sub-stoichiometric ratios with a distinct surplus of ethene. Although the flame temperature at this ratio should be somewhat higher compared to λ = 1, there is less formation of oxides. Apparently, the process needs a certain degree of reducing effect to overcome oxidative reactions promoted by the gas mixture itself and the surrounding air. Figure 17 shows the Ag coating at a higher resolution in SEM. It corresponds to the coating shown in Figure 16 in the fourth row, sprayed with 90 mm spray distance. However, in SEM there is no evidence for the presence of oxides. Figure 17b reveals the fine micropore structure that can be found in the coatings.

The coatings sprayed at closer distances and with a sub-stoichiometric oxygen-ethene-ratio are dense and no inter-layer porosity is visible. In contrast to the NiCr and copper coatings, the silver coatings show a pronounced correlation at sub-stoichiometric parameters between spraying distance and coating thickness.

Considering the high costs of silver powder feedstocks, deposition of thin coatings with high efficiency is an interesting approach. With a coating thickness of up to 30 µm deposited in four torch passes, the HVSFS process allows for a further reduction of the coating thickness. With 60 mm spray distance, it seems possible to perform 10 µm thick coatings in one torch pass. However, the short spraying distances result in an increased thermal load of the sample. Depending on the substrate and the desired coating thickness, a more elaborate cooling concept is needed.

**Figure 18:**
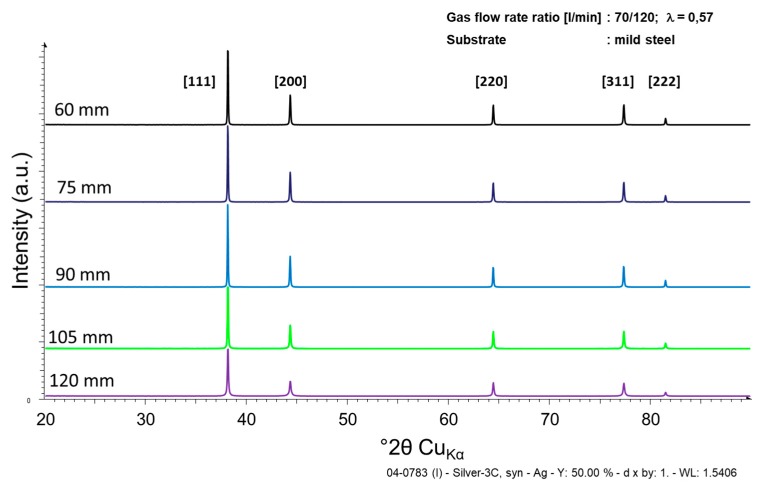
XRD spectrum of HVSFS sprayed Ag coatings as a function of spray distance (assigned as 60, 75, 90, 105, and 120 mm in the graph). Data correspond to samples in Figure 15, second row (60, 90, 120 mm).

Comparative XRD spectra are shown in Figure 18 for coatings sprayed with a gas flow rate of 70/120 and varying spray distances (60–120 mm; refer to Figure 15, second row). Only the peaks of pure silver are detectable, with peak intensity increasing towards lower spray distances. It was not possible to certainly identify oxide species in the XRD spectra.

**Figure 19:**
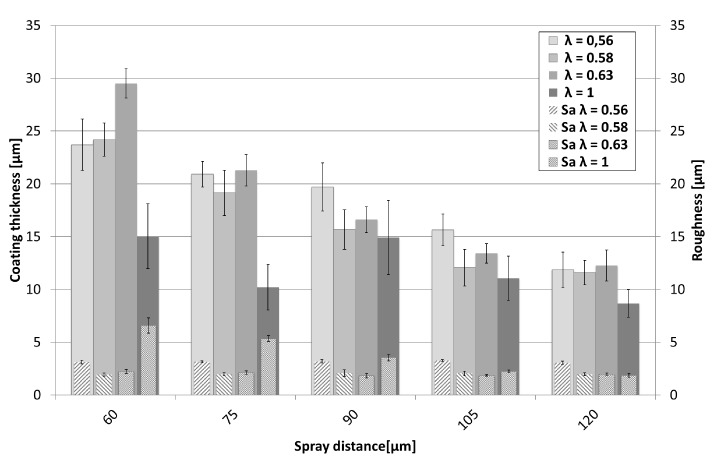
Comparison of coating thickness and roughness values of Ag coatings sprayed at different spray distances and λ values.

**Figure 20:**
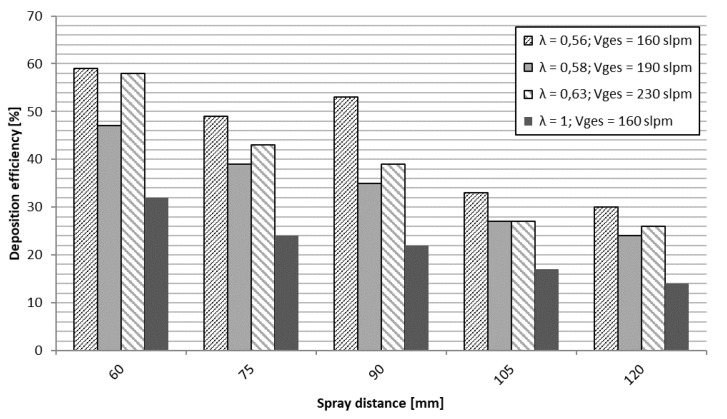
Deposition efficiency of Ag coatings for various spray parameter sets. Different spray distances for given sets of λ and total gas volume (slpm).

Achieved coating thickness and coating surface roughness show a clear dependency of spray distance and the ethene-oxygen ratio λ, as can be seen in Figure 19. Best coating structure and highest coating thickness was reached with sub-stoichiometric values (λ in the range of 0.56–0.63). The achieved coating thickness was in the range of 15–30 µm, sprayed with four torch passes, refer to Figure 19. Shorter spraying distances lead to higher deposition rates and accordingly demonstrate a higher deposition efficiency, as illustrated in Figure 20. Surface roughness on the other hand stays around 2 µm as can be seen in Figure 19. This is remarkable and may be due to the fact, that the heavy particles do not change their velocity significantly due to their high inertia at higher spray distances.

Spraying with stoichiometric parameter (λ = 1) on the other hand leads to the lowest deposition rate/efficiency and highest roughness values. The high roughness is mainly explained by the formation of defects on the coating surface as already discussed. This can be also clearly observed from the cross-section in Figure 16 (third row). Apparently, this parameter set is unsuitable to deposit defect free and smooth coatings. So far, the role of oxide formation at higher spray distances could not be clearly resolved in this study.

## 4. Conclusions and Outlook

In this study, suspensions containing metal powders of NiCr 80/20, copper and silver, suitable for the HVSFS process, were successfully prepared, characterized, and sprayed. Through variation of ethene and oxygen gas flow as well as the spraying distance, a suitable parameter window for each material could be established. The aim to spray dense and thin coatings with low oxidation rate and high metal content was fully achieved for silver and at least partially for copper. The results can open up interesting possibilities for electrical applications. Thin silver coatings may also serve as antibacterial surfaces. However, reduced spraying distances are necessary to achieve defect free silver coatings with reasonable deposition efficiencies. To minimize heat flux to the substrate, a different torch type or modified torch concept with reduced thermal power may be advantageous. In a future study, an in depth characterization regarding electrical conductivity together with the role of oxide formation, should be carried out in more detail.

The NiCr coatings show a fine dispersion of metal and oxide phases. Microstructure and oxidation are less affected by the spraying distance in comparison to silver and copper but can be adjusted by a variation of the gas flow. Due to its fine and evenly distributed oxide and the possibility to adjust coating thickness down to 10 µm or even lower, suspension sprayed NiCr can be an interesting approach for the improvement in film heating applications. This approach has been described and published recently by some of the authors [26].

Suspension preparation for metal powders on the other hand stays as a demanding task. High solid content and a long-term stability is overall difficult to achieve. As long as sedimentation occurs without the forming of stable aggregates, the suspension can stay processible by means of agitation and redispersion. This was especially the case for the suspensions used in this study. To reach industrially appropriate suspensions however, further development is necessary at this point.

## Figures and Tables

**Table 1:** Overview of the commercial powders used for suspension preparation. All data in this table are according to supplier datasheets.

	NiCr 80/20	Cu	Ag
Manufacturer	H.C. Starck	Evochem GmbH	Metalor Technologies SA
Notation	Amperit 251.051	14/200	P747-35
Density [g/cm³]	8.31	8.93	10.49
D10 [µm]	4	-	1.8
D50 [µm]	7.8	-	6.2
D90 [µm]	13.6	-	20
Powder type	Gas atomized	Chemically derived	Agglomerated and sintered
Morphology	spherical and dense	irregular dendritic	spherical

**Table 2:** Overview of the HVSFS spraying parameters discussed in this paper.

Parameter	NiCr		Cu		Ag	
Normalized ethene-oxygen ratio λ	0.87	1	0.57/0.63	1	0.63	1
*TGFR (oxygen + ethene) (slpm)	180	200	190/230	230	230	160
**GFR oxygen (slpm)	130	150	120/150	58	150	120
**GFR ethene (slpm)	50	50	70/80	172	80	40
Spray distance variations (mm)	For all three materials: 60; 75; 90; 105; 120					
Meander offset (mm)	For all three materials: 3					
Torch speed (mm/s)	For all three materials: 500					
Length of combustion chamber (mm)	22	22	22	22	12	12
Suspension feed rate (g/min)	55		43		52	
Number of torch passes	8	8	8	8	4	4

*TGFR = Total gas flow rate; **GFR = Gas flow rate; slpm = standard liters per minute.

**Table 3:** Rietveld analysis taken from XRD spectrum in Figure 8.

Phase (Ref. No.)	Fraction (wt%)	Error (wt%)
Nickel (000-04-0850)	60	±3
Chrome (000-01-1250)	14	±1
Spinell Cr_2_NiO_4_ (000-47-1049)	22	±3
Bunsenit NiO (000-75-0198)	4	±1,5

**Table 4:** EDX-Cu and O wt% values achieved from EDX on the samples shown in Figure 13.

Ref. No (Refer to Figure 13)	Cu (wt%)	O (wt%)	Other (wt%)
1	95.32 ± 0.6	0.71 ± 0.13	3.97 ± 0.31
2	87.79 ± 0.45	7.46 ± 0.10	5.74 ± 0.19
3	95.76 ± 0.56	0.69 ± 0.07	3.55 ± 0.27
4	92.66 ± 0.44	0.85 ± 0.04	6.49 ± 0.42
5	93.09 ± 0.47	6.91 ± 0.11	-
6	95.11 ± 0.47	4.63 ± 0.1	0.25 ± 0.18
7	97.09 ± 0.45	2.64 ± 0.09	0.27 ± 0.04
8	95.11 ± 0.48	4.89 ± 0.10	-
9	97.06 ± 0.48	2.94 ± 0.09	-
